# Safety and Efficacy of Ultrasound-Guided Retrolaminar and Midpoint Transverse Process to Pleura Blocks Compared to Ultrasound-Guided Classic Paravertebral Block for Breast and Thoracic Surgery: A Systematic Review and Meta-Analysis

**DOI:** 10.7759/cureus.92777

**Published:** 2025-09-20

**Authors:** Raphael Matheus de Souza Makiyama Lopes, Priscila Ferreira de Lima e Souza, Gustavo Gohringer de Almeida Barbosa, Anisio Uchoa Leite Santana, Cecília Schettini Gueiros, Matheus Requena Escobar, João Evangelista Ponte Conrado, Antonio Andrea Camastra, Lucas Teixeira Baldo, André Busatto de Donato, Laiz G. C. Novaes, Daniel Macedo Oliveira, Thomas Rolf Erdmann

**Affiliations:** 1 Department of Anesthesiology, Hospital Unimed Balneário Camboriú, Balneário Camboriú, BRA; 2 School of Medicine, Universidade Federal do Ceará, Fortaleza, BRA; 3 Department of Orthopedic Surgery, Hospital Divina Providência, Porto Alegre, BRA; 4 School of Medicine, Universidade de São Paulo, São Paulo, BRA; 5 School of Medicine, Faculdade Pernambucana de Saúde, Recife, BRA; 6 Department of Anesthesiology, Centro Universitário Lusíada, Santos, BRA; 7 Department of Anesthesiology, Università degli Studi "Magna Græcia" di Catanzaro, Catanzaro, ITA; 8 Department of Anesthesiology, Instituto de Cirurgia do Lago, Brasília, BRA; 9 Department of Anesthesiology, Universidade de São Paulo, São Paulo, BRA; 10 School of Medicine, Universidade Estácio de Sá, Rio de Janeiro, BRA; 11 School of Medicine, Universidade Federal da Paraíba, João Pessoa, BRA; 12 Department of Surgery, Universidade Federal de Santa Catarina, Florianópolis, BRA

**Keywords:** breast surgery, paravertebral block, postoperative analgesia, regional anesthesia, thoracic surgery

## Abstract

The ultrasound-guided midpoint transverse process to pleura (MTP) block and retrolaminar block (RLB) have emerged as promising alternatives to the classic paravertebral block (PVB) for breast and thoracic surgery. This systematic review and meta-analysis of randomized controlled trials was conducted to compare the analgesic efficacy and safety of these newer techniques with the traditional PVB. Our pooled analysis revealed that MTP block and RLB offer early postoperative analgesic efficacy comparable to PVB. However, the MTP block/RLB group tended to have slightly higher pain scores with movement and greater cumulative opioid consumption in the first 24 hours. Regarding safety, all reported cases of pneumothorax or pleural puncture occurred exclusively in the PVB group. The enhanced safety profile and potentially simpler technique associated with the MTP block and RLB suggest that they may be valuable alternatives to the classic PVB in clinical practice.

## Introduction and background

Breast and thoracic surgeries are routinely performed worldwide, requiring effective perioperative pain control to facilitate recovery and enhance patient comfort. Modern guidelines emphasize multimodal, opioid-sparing analgesia to enhance recovery, minimize opioid-related adverse effects such as postoperative nausea and vomiting (PONV), and improve overall clinical outcomes [1,2]. Within this framework, regional anesthesia plays a central role.

The paravertebral block (PVB) has long been regarded as a reliable option for thoracic and breast surgery analgesia. Indeed, the origins of the PVB date back more than a century, with a seminal description of its utility for thoracic procedures appearing as early as 1922 in Gaston Labat's foundational work "Regional Anesthesia" [3]. In the modern era, multiple systematic reviews have demonstrated efficacy comparable to the traditional gold standard, thoracic epidural analgesia (TEA) [4-6]. The Procedure-Specific Postoperative Pain Management (PROSPECT) guidelines specifically recommend PVB for patients undergoing video-assisted thoracoscopic surgery (VATS) and as the first-line regional technique for major oncologic breast surgery, integrated into a multimodal analgesic regimen [7]. Despite being a well-established procedure, it presents technical challenges, requires significant operator experience, and carries risks such as pneumothorax and vascular punctures [8].

A precise understanding of the thoracic paravertebral space (TPVS) anatomy is essential for comprehending the mechanism of action of paraspinal blocks and their clinical applications. The TPVS is a wedge-shaped anatomical space located alongside the thoracic vertebral column and lateral to the vertebral body. Its boundaries are the parietal pleura and endothoracic fascia anterolaterally; the vertebral body, intervertebral foramen, and intervertebral disc medially; the transverse processes, ribs, and superior costotransverse ligament (SCTL) posteriorly; and the psoas muscle at the L1 level caudally [9,10]. The SCTL acts as a porous posterior border for the TPVS. This incomplete barrier between the internal intercostal membrane and the vertebral body allows for extensive connection between the TPVS and retro-SCTL space through various openings, explaining the effectiveness of certain block techniques that do not directly access the TPVS. The efficacy of the midpoint transverse process to pleura (MTP) block relies on the anesthetic spreading into the paravertebral space, which is hypothesized to occur via two primary routes: directly through fenestrations within the SCTL or medially around the ligament's free edge [11]. The retrolaminar block (RLB) was described in 2006 and involves the deposition of a local anesthetic into the fascial plane between the posterior surface of the thoracic lamina and the overlying transversospinalis muscles [12]. Cadaveric evidence has confirmed that the solution spreads anteriorly through the intertransverse ligaments to access both the paravertebral and epidural spaces [13].

The advent of ultrasound has fundamentally transformed regional anesthesia, enabling the rapid development of novel fascial plane blocks [14]. It facilitates drug deposition adjacent to the paravertebral space without direct penetration, producing a paravertebral by proxy effect [15]. Emerging evidence, including recent randomized controlled trials (RCTs), suggests that certain ultrasound-guided fascial plane techniques, particularly the MTP block and RLB, may provide similar analgesic benefits with a potentially improved safety profile and simpler execution [16-20]. This review aims to consolidate and critically evaluate the available evidence on MTP block and RLB, assessing their comparative analgesic effectiveness and safety profiles with those of PVB.

## Review

Methods

Protocol and Registration

We performed this systematic review and meta-analysis in accordance with the Cochrane Handbook for Systematic Reviews of Interventions and the Preferred Reporting Items for Systematic Reviews and Meta-Analyses (PRISMA) statement guidelines [21,22]. The study protocol was registered in the International Prospective Register of Systematic Reviews (PROSPERO) under the registration number CRD420251038396.

Eligibility Criteria

The PICO question guiding this study was as follows: In patients undergoing breast or thoracic surgery, does the application of RLB or MTP block lead to enhancements in pain management, a reduction in opioid requirements, and a lower incidence of complications when compared with the classic PVB?

We included RCTs involving adult patients undergoing thoracic or breast surgery that compared either RLB or MTP block with classic ultrasound-guided PVB. Studies were required to report at least one of our specified outcomes. We excluded non-English publications and studies with unrelated patient populations, interventions, and outcomes.

Search Strategy and Study Selection

We conducted searches across the Cochrane Library, PubMed, and Embase databases until May 2025. The search strategy was performed, including relevant keywords, Boolean operators, and specific search strings for each database (Medical Subject Headings (MeSH)/Emtree terms). Key search terms included "paravertebral block", "retrolaminar block", "midpoint transverse process block", "thoracic surgery", "breast surgery", and "postoperative pain".

The screening process was independently conducted by two authors using the Rayyan software (Rayyan Systems Inc., Cambridge, Massachusetts, United States). The third author adjudicated conflicts regarding the study inclusion. We attempted to contact the authors via email to obtain additional information and data but were unsuccessful.

Data Extraction and Management

Data collection was organized in a standard spreadsheet, including study characteristics (author, publication year, sample size, study design, patient characteristics (age, body mass index (BMI)), surgical procedure, block characteristics, and all relevant outcomes). The data are summarized below. The identified publications were evaluated to retrieve data regarding the variables relevant to the outcomes under investigation. The primary outcome was postoperative pain scores at one, six, 12, and 24 hours using the visual analog scale (VAS) or numeric rating scale (NRS). Secondary outcomes included cumulative opioid consumption (mg of morphine equivalents) at 24 hours postoperatively, block performance time, time to request rescue analgesia, and incidence of complications (hypotension, pneumothorax, pleural puncture, and PONV). Data were primarily extracted from the tables and text. When data were presented as graphs or figures, WebPlotDigitizer (Ankit Rohatgi) was used to extract the numerical values. If data were reported as median and interquartile range, we estimated the mean and standard deviation using the calculator tool by Wan et al. and Luo et al. [23,24]. This calculator also assesses the skewness of the data distribution, and for significantly skewed data distributions, the mean and standard deviation are not calculated. In this situation, we did not include the specific outcome data from that study in the statistical analysis.

Risk of Bias Assessment

The methodological quality of each RCT was assessed independently by two reviewers using the Cochrane Risk of Bias tool (RoB 2) [25]. This tool evaluates five domains of bias: bias from the randomization process, bias due to deviations from intended interventions, bias due to missing outcome data, bias in the measurement of the outcome, and bias in the selection of the reported result. The risk of bias was determined as "high risk", "some concerns", and "low risk". In cases of disagreement, a third reviewer was consulted to resolve the discrepancies.

Data Synthesis and Statistical Analysis

Review Manager (RevMan v.5.4, The Cochrane Collaboration, London, England, United Kingdom) was used to perform the meta-analysis. Statistical analysis with forest plots was performed only for outcomes for which a minimum of three trials contributed data. For continuous data, the mean difference (MD) and standard deviation were used, along with a 95% CI. We used the inverse variance method to calculate the MDs between the groups. Postoperative opioid consumption was evaluated by extracting the opioid dose data reported in each of the included studies. To enable standardized comparisons across studies using different opioids, all opioid doses were converted to oral morphine equivalent (OME) doses. Opioid dose conversions were performed using the ClinCalc online opioid conversion calculator [26]. When studies reported the time to the first postoperative analgesic request in minutes, values were converted to hours by dividing both the mean and standard deviation by 60. For dichotomous data, risk ratios and 95% CIs were calculated. A random-effects model was implemented to manage potential heterogeneity, estimating the between-study variance using the restricted maximum likelihood approach. Heterogeneity was quantified using the I² statistic, and all results were reported with corresponding 95% CI. To explore potential sources of heterogeneity in outcomes where the I² statistic exceeded 50%, a sensitivity analysis was performed using the R software (R Foundation for Statistical Computing, Vienna, Austria). We conducted a subgroup analysis based on block type (RLB vs. MTP block) to explore the influence of the different block techniques on the outcomes. Funnel plots were used to evaluate publication bias for primary outcomes. Finally, the overall quality of evidence for the primary outcomes was assessed using the Grading of Recommendations, Assessment, Development, and Evaluation (GRADE) methodology, which rates evidence as high, moderate, low, or very low based on factors such as risk of bias, inconsistency, imprecision, and indirectness [27].

Results

Study Selection

**Figure 1:**
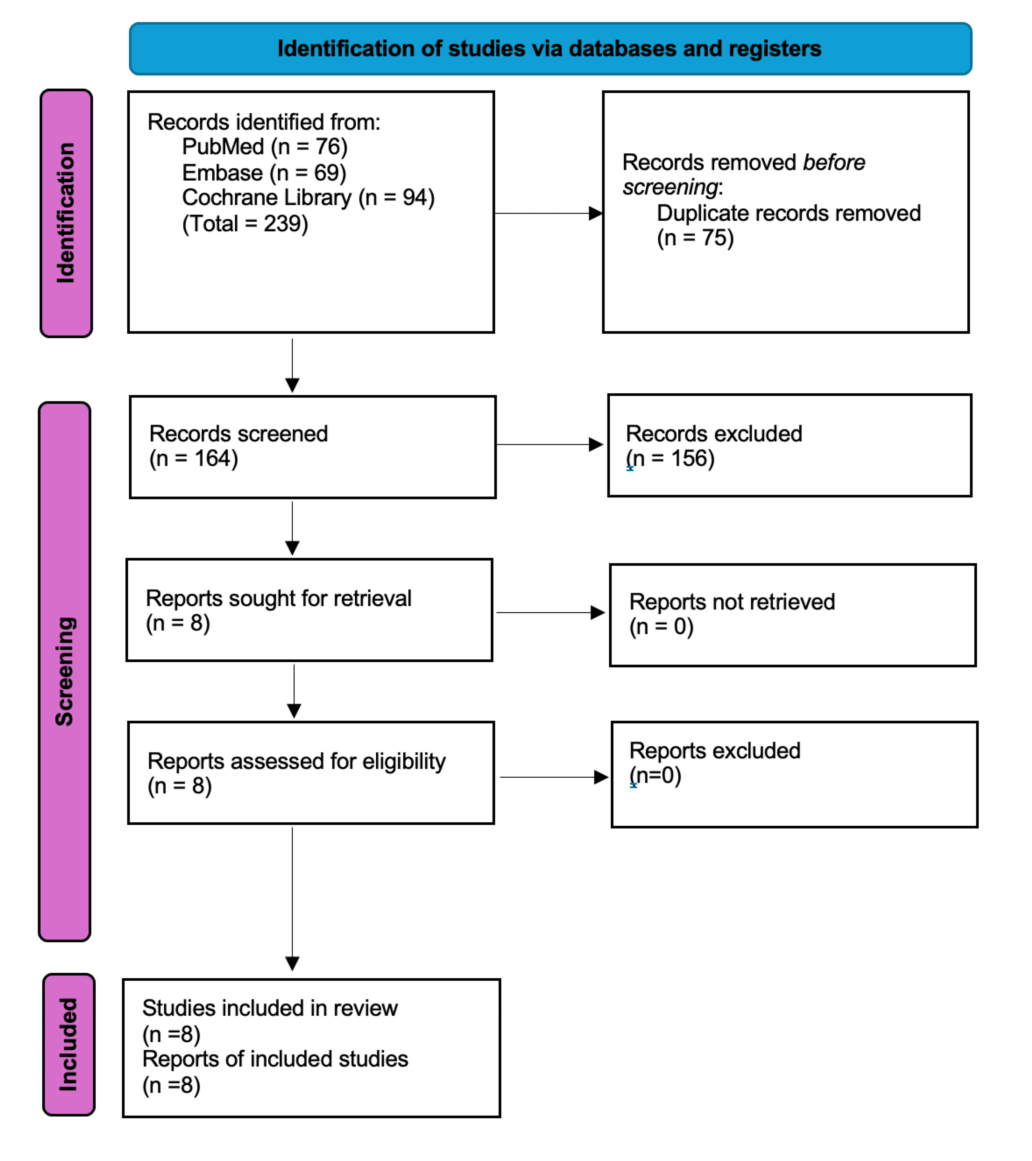
PRISMA flow diagram of study selection Included reports consist of seven full-text articles and one conference abstract. PRISMA: Preferred Reporting Items for Systematic Reviews and Meta-Analyses

The study selection process is illustrated in the PRISMA flow diagram (Figure 1). A total of 239 records were identified through database searches. After removing 75 duplicate records, 164 records were screened based on their titles and abstracts. Eight studies were assessed for eligibility, and their full texts were reviewed. Finally, we retrieved seven full-text articles and one abstract from the congress. Eight RCTs with a total of 483 patients were included in this meta-analysis [16-20,28-30].

Study Characteristics

The characteristics of the included studies are shown in Table 1. All studies were RCTs published between 2021 and 2024 [16-20,28-30]. Four studies compared PVB with MTP block [16-18,28], and four compared PVB with RLB [19,20,29,30]. Across all studies, 240 patients received PVB, and 243 patients received MTP block or RLB. BMI was homogeneously distributed within the normal to overweight range across the studies. The surgical procedures included in these studies were VATS and breast surgery. Most studies used a single injection technique, except for one study (Wang et al. [30]), followed by patient-controlled intravenous analgesia (PCIA) for postoperative pain management. The study by Swathi et al. was an exception, in which a catheter was inserted [17]. The local anesthetic was ropivacaine or bupivacaine with a concentration between 0.20% and 0.5% and a volume between 10 ml and 30 ml. Only Singh et al. added dexmedetomidine to the local anesthetic [28]. Table 1 details the postoperative analgesia regimen, block performance level, number of injections, and volume and concentration of local anesthetic used in each study.

**Table 1:** Characteristics of the included studies ᵃNA: not available ᵇData presented as median (interquartile range) ᶜExact thoracic level not specified ᵈBlock performed at multiple levels between T3 and T5 BMI: body mass index; IV: intravenous; MTP: midpoint transverse process to pleura; NSAIDs: nonsteroidal anti-inflammatory drugs; PCIA: patient-controlled intravenous analgesia; PVB: paravertebral block; RLB: retrolaminar block; SITS: single-incision thoracoscopic surgery; uVATS: uniportal video-assisted thoracoscopic surgery; VATS: video-assisted thoracoscopic surgery

Characteristic	Group	Chen et al., 2022 [16]	Swathi et al., 2021 [17]	Kahramanlar et al., 2022 [18]	Gao et al., 2023 [19]	Zhu et al., 2025 [20]	Singh et al., 2024 [28]	Sugiyama et al., 2021 [29]	Wang et al., 2021 [30]
Type of study	-	RCT	RCT	RCT	RCT	RCT	RCT	RCT	RCT
Total number	MTP block	41	18	32	-	-	40	-	-
	RLB	-	-	-	30	27	-	25	30
	PVB	40	18	32	30	25	40	25	30
BMI (kg/m²)	MTP block	23.6±3.2	21.6±3.1	28.7±3.0	-	-	NAᵃ	-	-
	RLB	-	-	-	24.4±3.7	22.0±3.2	-	21 (20-23)ᵇ	23.2±3.5
	PVB	23.0±2.9	21.4±3.1	28.9±3.1	22.5±2	22.2±3.9	NAᵃ	NAᵃ	24.3±3.0
Age (years)	MTP block	59.8±11.5	36.5±11.1	51.0±5.7	-	-	NAᵃ	-	-
	RLB	-	-	-	56.0±13.1	50.6±12.2	-	66 (31-73)ᵇ	55.3±11.8
	PVB	58.4±10.5	31.2±12.2	52.6±6.0	59.2±11.8	50.3±14.58	NAᵃ	NAᵃ	53.7±14.0
Type of surgery	-	uVATS	VATS	Unilateral mastectomy	VATS	SITS	Modified radical mastectomy	VATS or limited thoracotomy	uVATS
Block level/region	-	Thoracic T5/T6	Thoracic T4/T5	Thoracic T3/T4	Thoracic T6	Thoracicᶜ	Thoracicᵈ	Thoracic T6	Thoracic T3/T5ᶜ
Local anesthetic	-	15 mL of 0.5% ropivacaine	20 mL of 0.2% ropivacaine, catheter	20 mL of 0.25% bupivacaine	20 mL of 0.375% ropivacaine	20 mL of 0.5% ropivacaine	20 mL 0.5% bupivacaine+0.6 µg/kg dexmedetomidine	RLB (40 mL 0.5% ropivacaine), PVB (20 mL 0.5% ropivacaine)	30 mL of 0.5% ropivacaine
Postop. analgesia	-	PCIA (sufentanil, flurbiprofen)	Continuous block infusion+rescue analgesia	PCIA (fentanyl)+IV paracetamol	PCIA (tramadol, lornoxicam)	PCIA (sufentanil)+rescue NSAIDs	NAᵃ	PCIA (morphine)+IV/oral NSAIDs	PCIA (sufentanil)

Risk of Bias and Publication Bias

**Figure 2:**
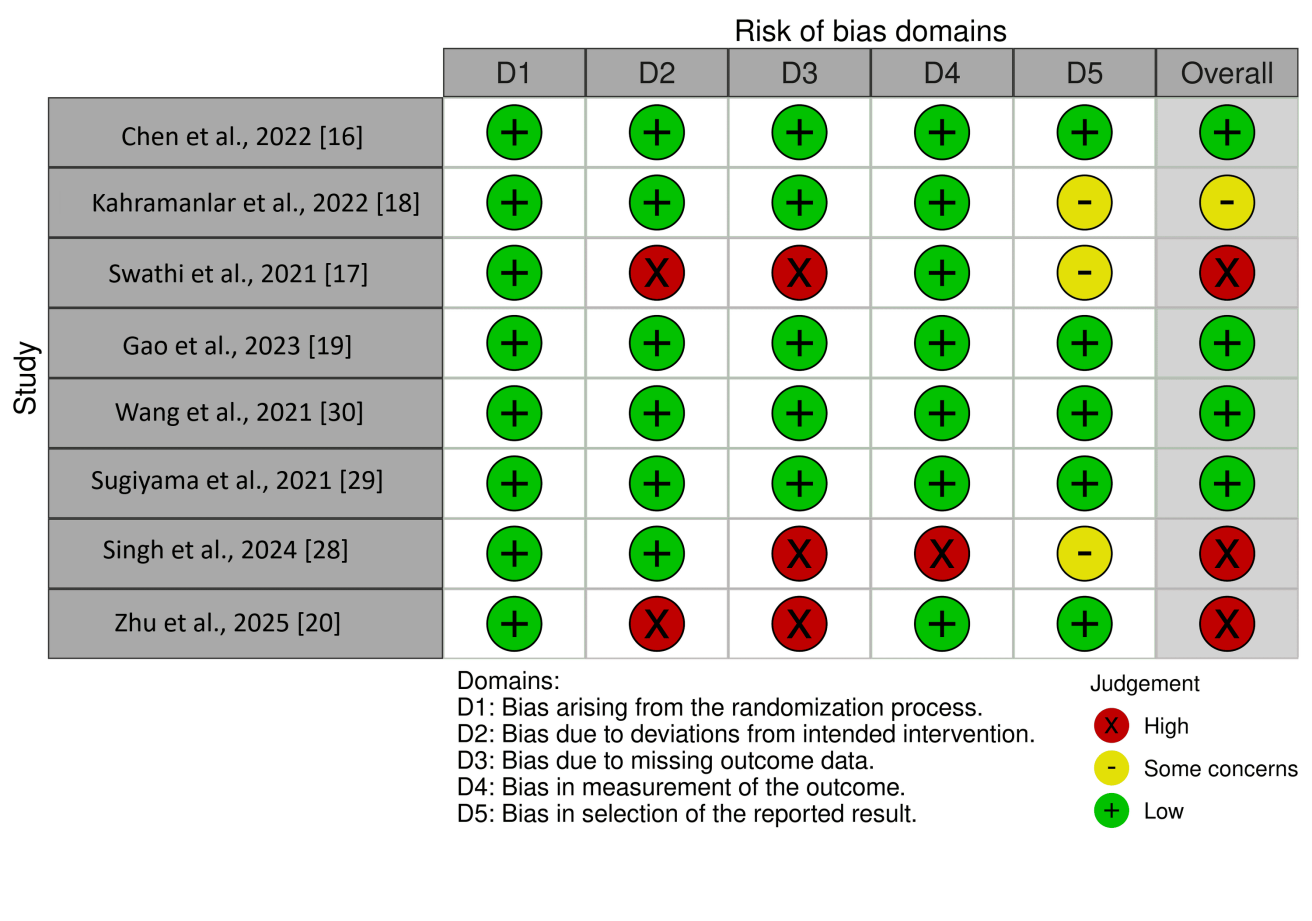
Cochrane risk of bias assessment of randomized controlled trial for each individual study (ROB 2)

The overall risk of bias assessed using the ROB 2 tool (Figure 2) was considered low in most studies [16,18,19,29,30] and high in three studies [17,20,28]. We assessed publication bias using funnel plots for primary outcomes. Despite the creation of the funnel plot, the interpretation of the funnel plot can be subjective and was not sufficiently clear, probably because the number of included studies was fewer than 10. A proper Egger's test could not be conducted due to the number of included studies.

Primary Outcome: Postoperative Pain Scores at Rest and Movement at One, Six, 12, and 24 Hours After Surgery

**Figure 3:**
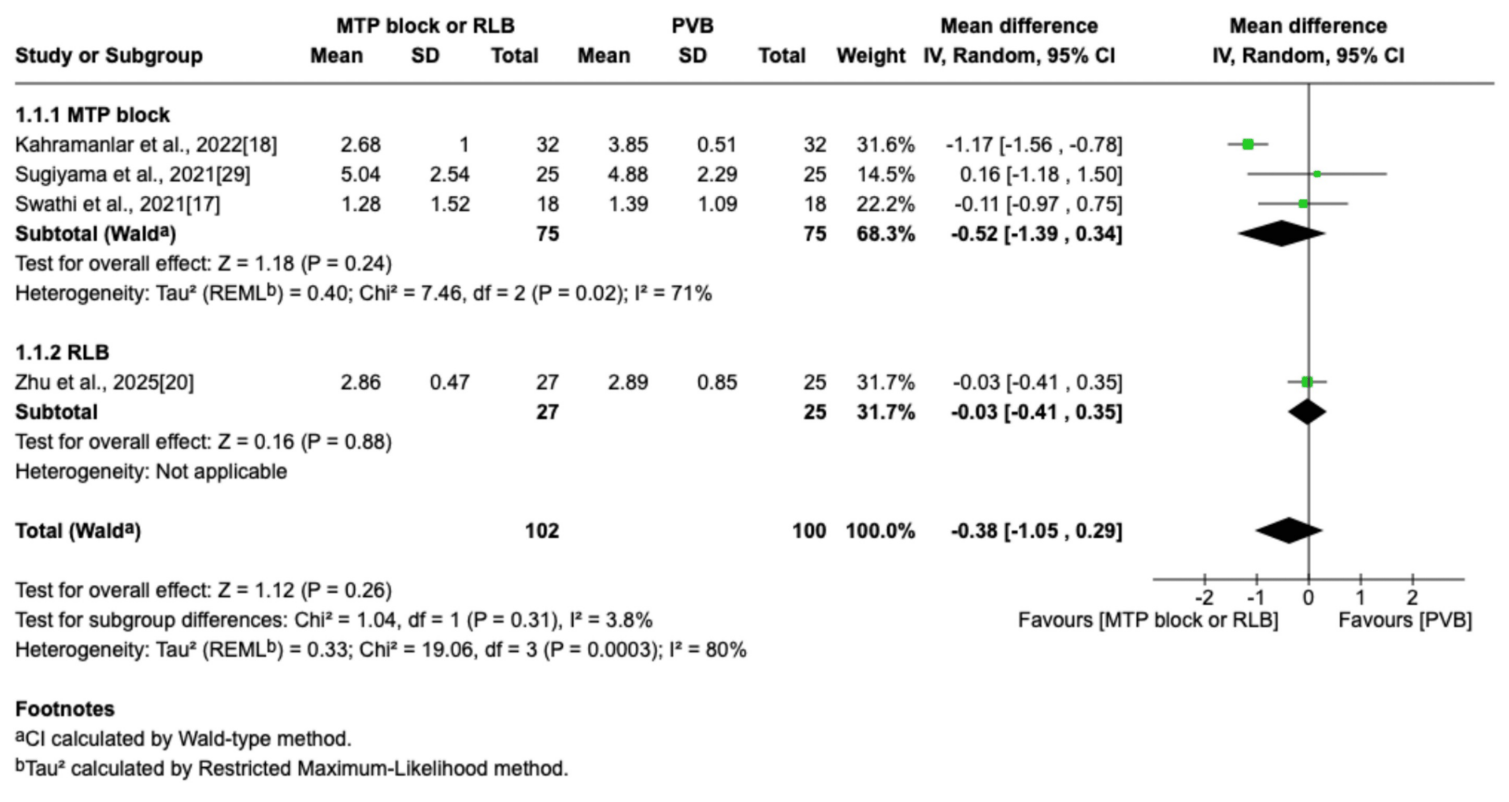
Forest plot of postoperative pain scores (one hour rest) MTP: midpoint transverse process to pleura; RLB: retrolaminar block; PVB: paravertebral block; MD: mean difference; SD: standard deviation

**Figure 4:**
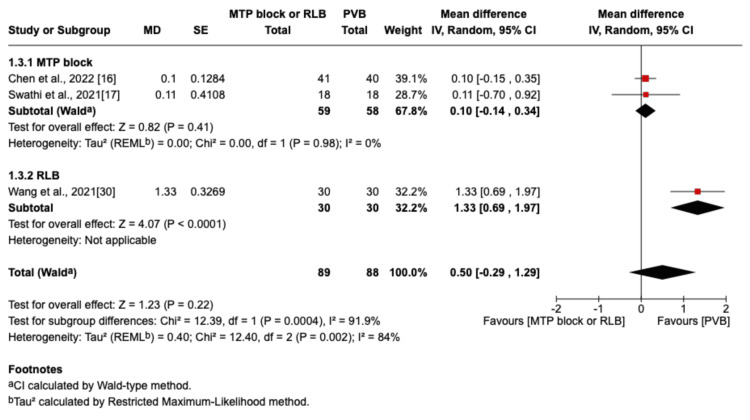
Forest plot of postoperative pain scores (six hours rest) MTP: midpoint transverse process to pleura; RLB: retrolaminar block; PVB: paravertebral block; MD: mean difference; SD: standard deviation

**Figure 5:**
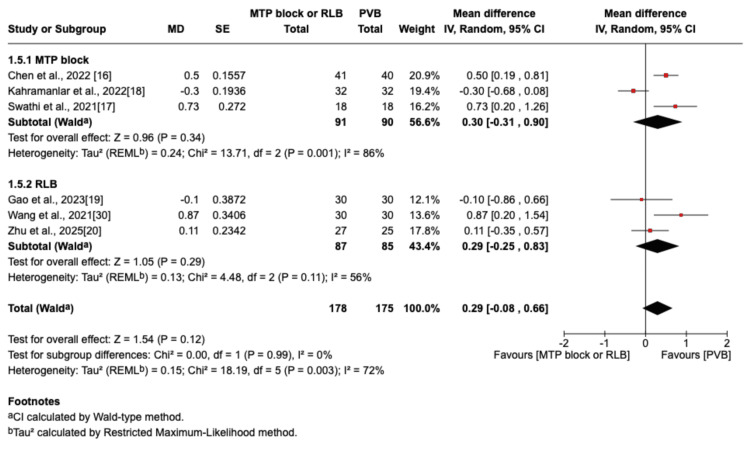
Forest plot showing postoperative pain scores (12 hours rest) MTP: midpoint transverse process to pleura; RLB: retrolaminar block; PVB: paravertebral block; MD: mean difference; SD: standard deviation

**Figure 6:**
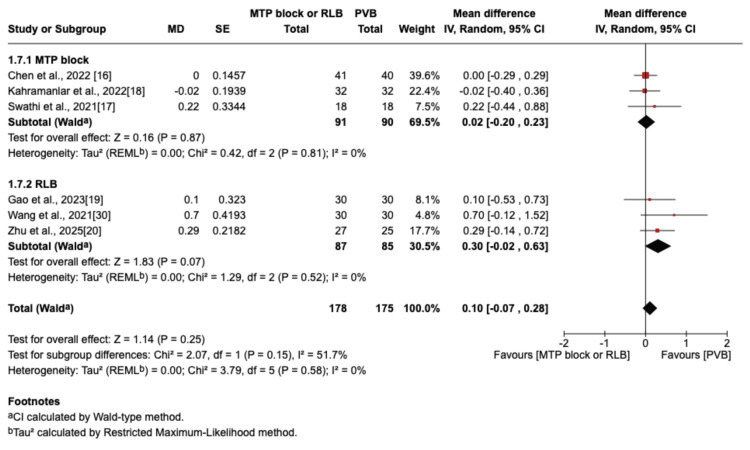
Forest plot showing postoperative pain scores (24 hours rest) MTP: midpoint transverse process to pleura; RLB: retrolaminar block; PVB: paravertebral block; MD: mean difference; SD: standard deviation

**Figure 7:**
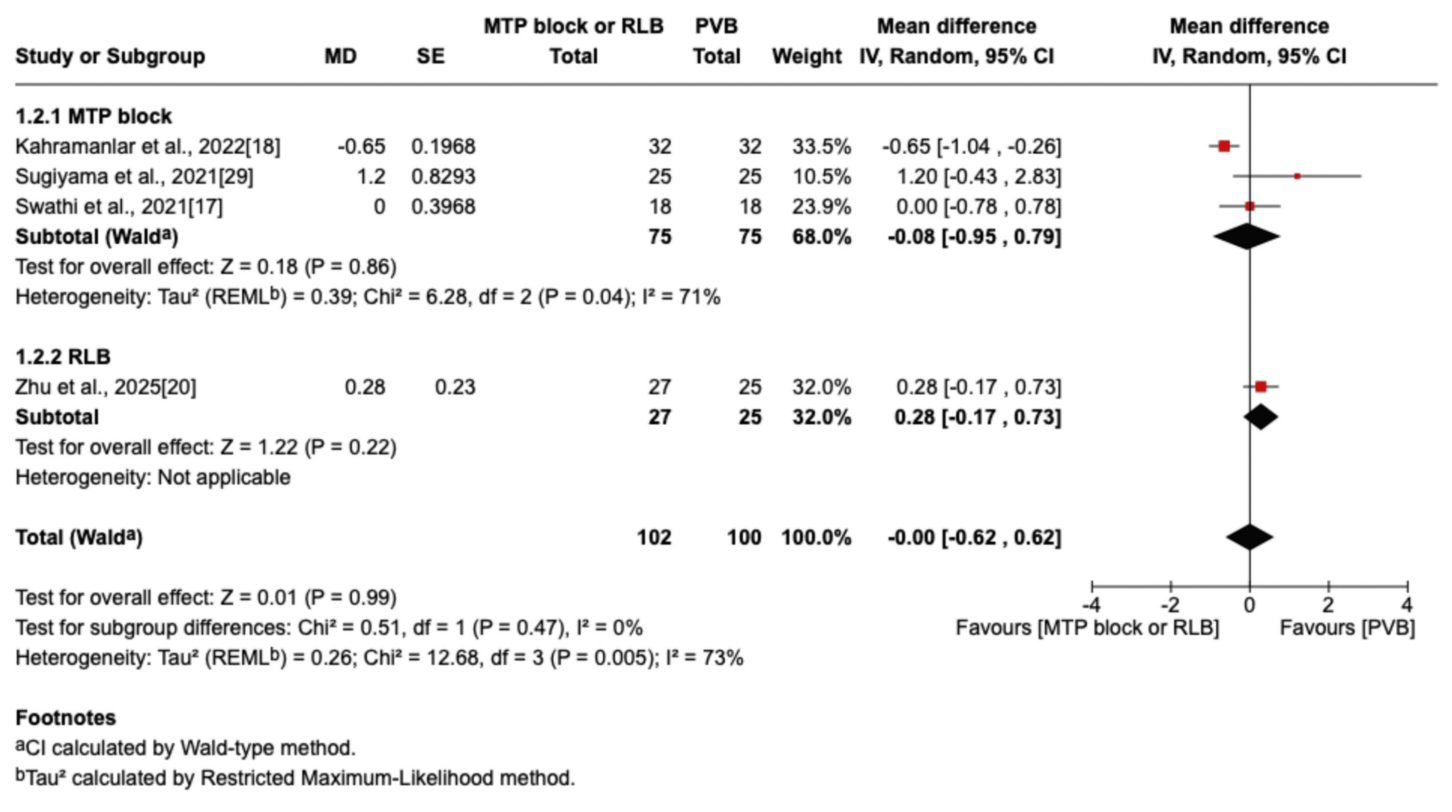
Forest plot of postoperative pain scores (one hour movement) MTP: midpoint transverse process to pleura; RLB: retrolaminar block; PVB: paravertebral block; MD: mean difference; SD: standard deviation

**Figure 8:**
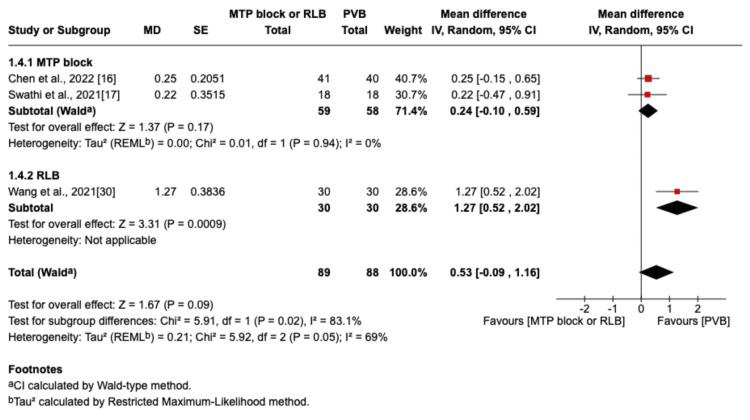
Forest plot of postoperative pain scores (six hours movement) MTP: midpoint transverse process to pleura; RLB: retrolaminar block; PVB: paravertebral block; MD: mean difference; SD: standard deviation

**Figure 9:**
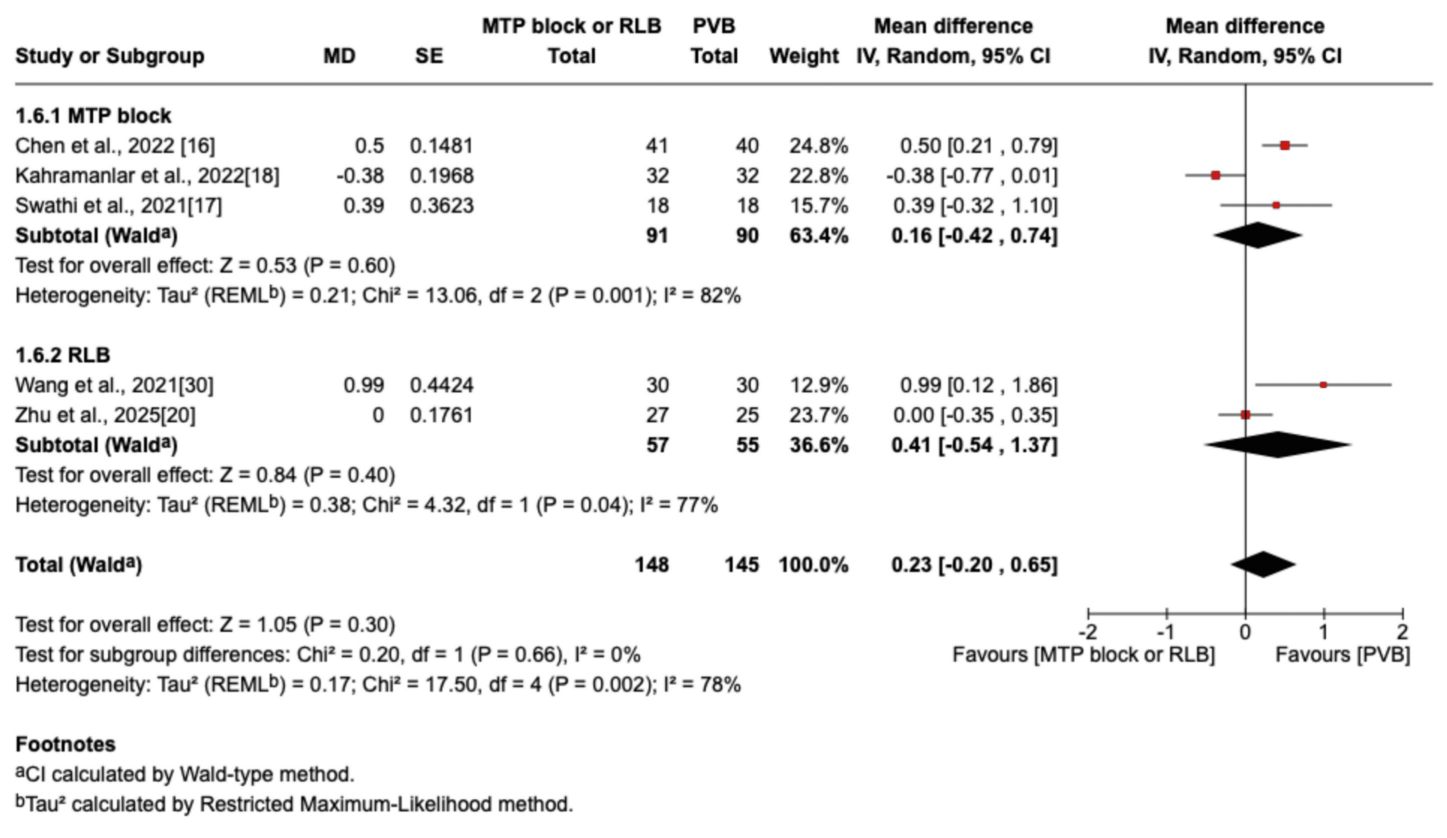
Forest plot showing postoperative pain scores (12 hours movement) MTP: midpoint transverse process to pleura; RLB: retrolaminar block; PVB: paravertebral block; MD: mean difference; SD: standard deviation

The outcome was reported by seven RCTs: six studies were in the thoracic surgery population and one was in the breast surgery population [16-20,29,30]. Of these seven studies, four compared RLB with PVB [19,20,29,30], and three compared MTP block with PVB [16-18]. Postoperative pain scores at rest were not significantly different between the PVB and MTP block/RLB groups at one hour (MD=-0.38; 95% CI=-1.05 to 0.29; p=0.26; I²=80%; Figure 3), six hours (MD=0.5; 95% CI=-0.29 to 1.29; p=0.22; I²=84%; Figure 4), 12 hours (MD=0.29; 95% CI=-0.08 to 0.66; p=0.12; I²=72%; Figure 5), or 24 hours (MD=0.10; 95% CI=-0.07 to 0.28; p=0.25; I²=0%; Figure 6). Similarly, there were no significant differences when comparing postoperative pain scores during movement at one hour (MD=0; 95% CI=-0.62 to 0.62; p=0.99; I²=73%; Figure 7), six hours (MD=0.53; 95% CI=-0.09 to 1.16; p=0.09; I²=69%; Figure 8), and 12 hours (MD=0.23; 95% CI=-0.20 to 0.65; p=0.30; I²=78%; Figure 9).

**Figure 10:**
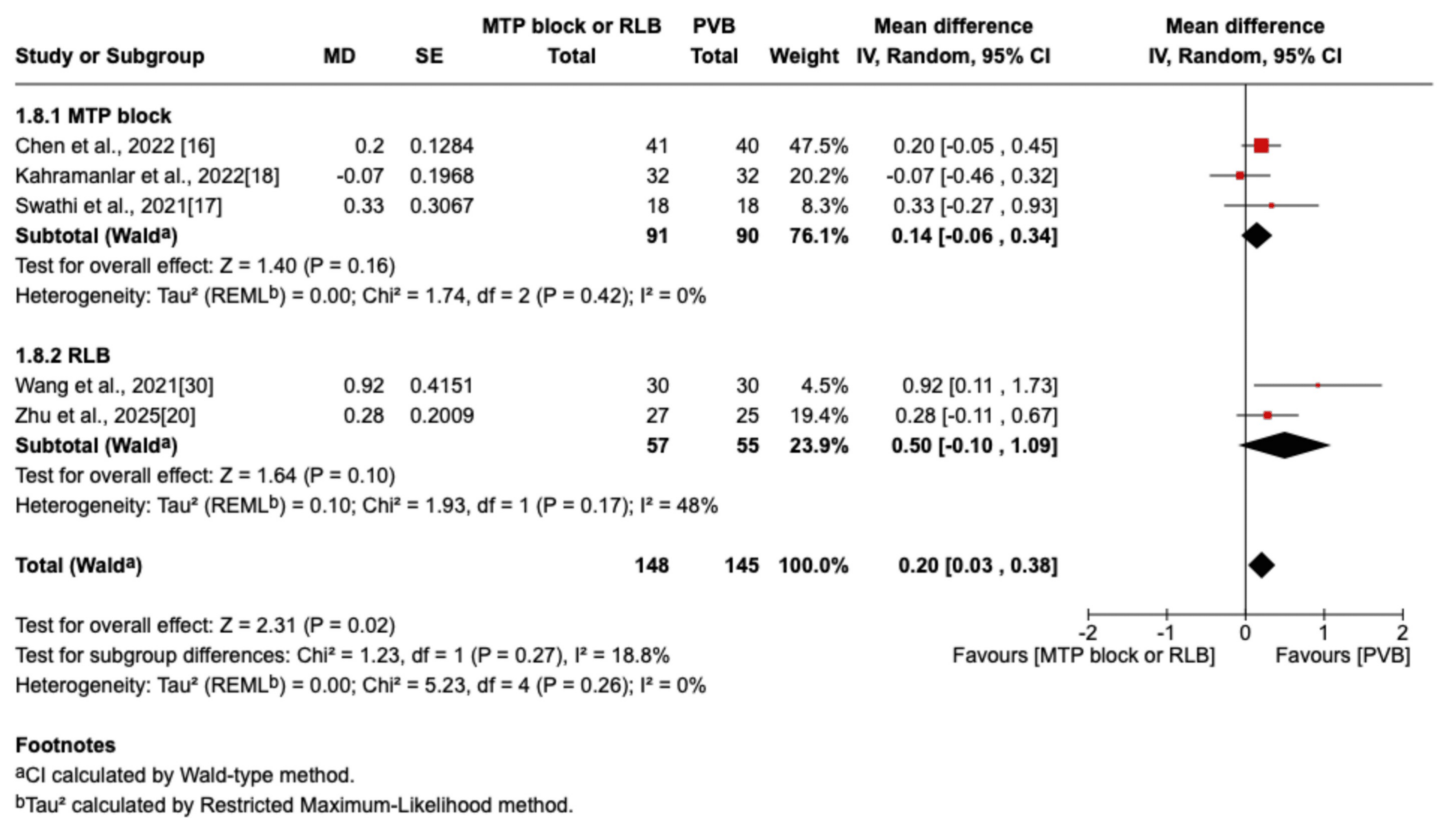
Forest plot showing postoperative pain scores (24 hours movement) MTP: midpoint transverse process to pleura; RLB: retrolaminar block; PVB: paravertebral block; MD: mean difference; SD: standard deviation

However, at 24 hours during movement, there was a significant difference in favor of the PVB group (MD=0.20; 95% CI=0.03 to 0.38; p=0.02; I²=0%; Figure 10). 

Cumulative Opioid Consumption at 24 Hours After Surgery

**Figure 11:**
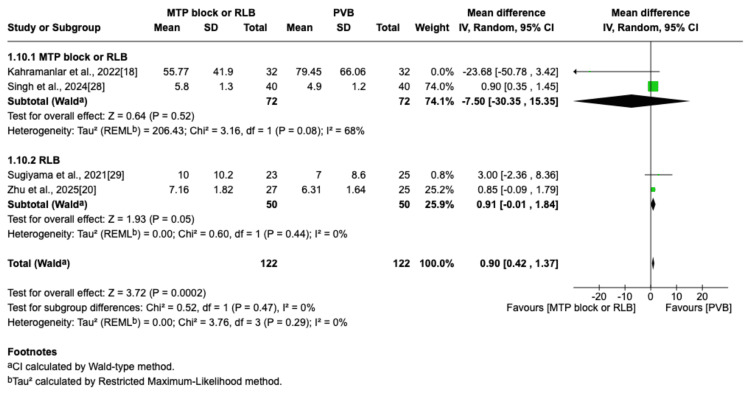
Forest plot of 24-hour postoperative opioid consumption (OME) OME: oral morphine equivalent; MTP: midpoint transverse process to pleura; RLB: retrolaminar block; PVB: paravertebral block; MD: mean difference; SD: standard deviation

This outcome was reported in four trials, including 244 patients (122 in the MTP block/RLB group and 122 in the PVB group) [18,20,28,29]. Across all four studies, postoperative pain was managed with PCIA, providing a standardized method for rescue analgesia. The specific opioids used in the PCIA regimens varied between studies and included fentanyl, sufentanil, and morphine, with some protocols also incorporating multimodal analgesia as detailed in Table 1. The forest plot indicates a slight increase in morphine consumption in the MTP block/RLB group compared to the PVB group (MD=0.90; 95% CI=0.42 to 1.37; p=0.0002; I^2^=0%; Figure 11). Despite a statistically significant increase in 24-hour opioid consumption observed in the MTP block/RLB group, this difference is unlikely to be clinically significant, as evidenced by a systematic review by Laigaard et al., which identified a minimal clinically important difference for postoperative opioid consumption of 10 mg of morphine equivalents [31].

Time to First Postoperative Analgesic Rescue (Hours)

**Figure 12:**
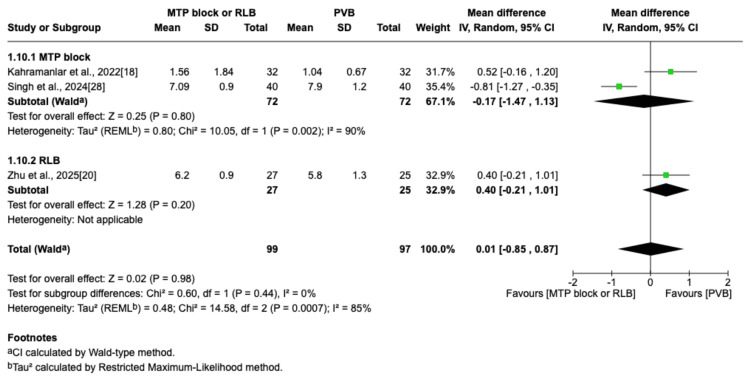
Forest plot of time to first postoperative analgesic rescue (hours) MTP: midpoint transverse process to pleura; RLB: retrolaminar block; PVB: paravertebral block; MD: mean difference; SD: standard deviation

This outcome was reported in three trials [18,20,28]. There was no statistically significant difference in the time to the first postoperative analgesic request between the groups (MD=0.01; 95% CI=-0.85 to 0.87; p=0.98; I^2^=85%; Figure 12).

Time to Perform Block (Minutes)

**Figure 13:**
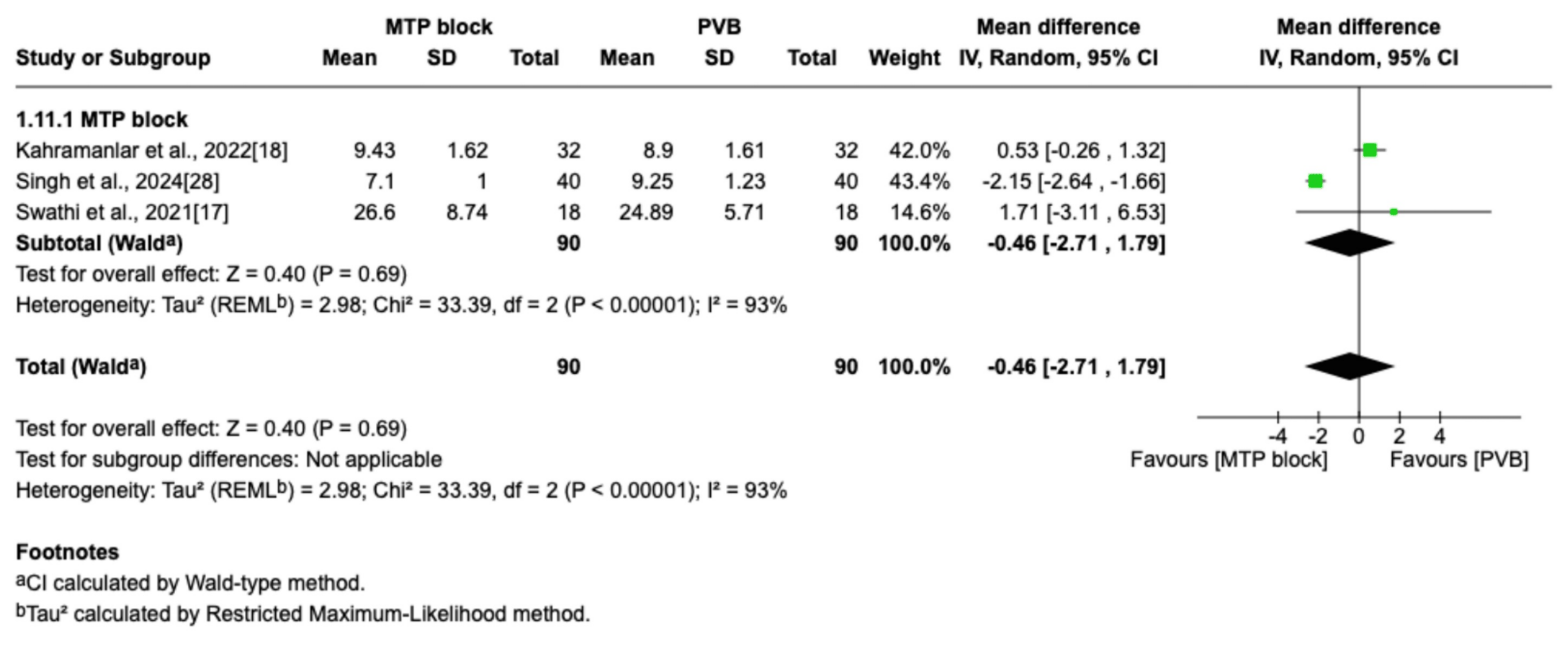
Forest plot of block performance time (minutes) MTP: midpoint transverse process to pleura; RLB: retrolaminar block; PVB: paravertebral block; MD: mean difference; SD: standard deviation

All three trials that reported this outcome compared the MTP block and PVB groups [17,18,28]. The pooled analysis did not reveal any significant difference between the two groups in the time required to complete the block (MD=-0.46; 95% CI=-2.71 to 1.79; p=0.69; I^2^=93%; Figure 13).

PONV

**Figure 14:**
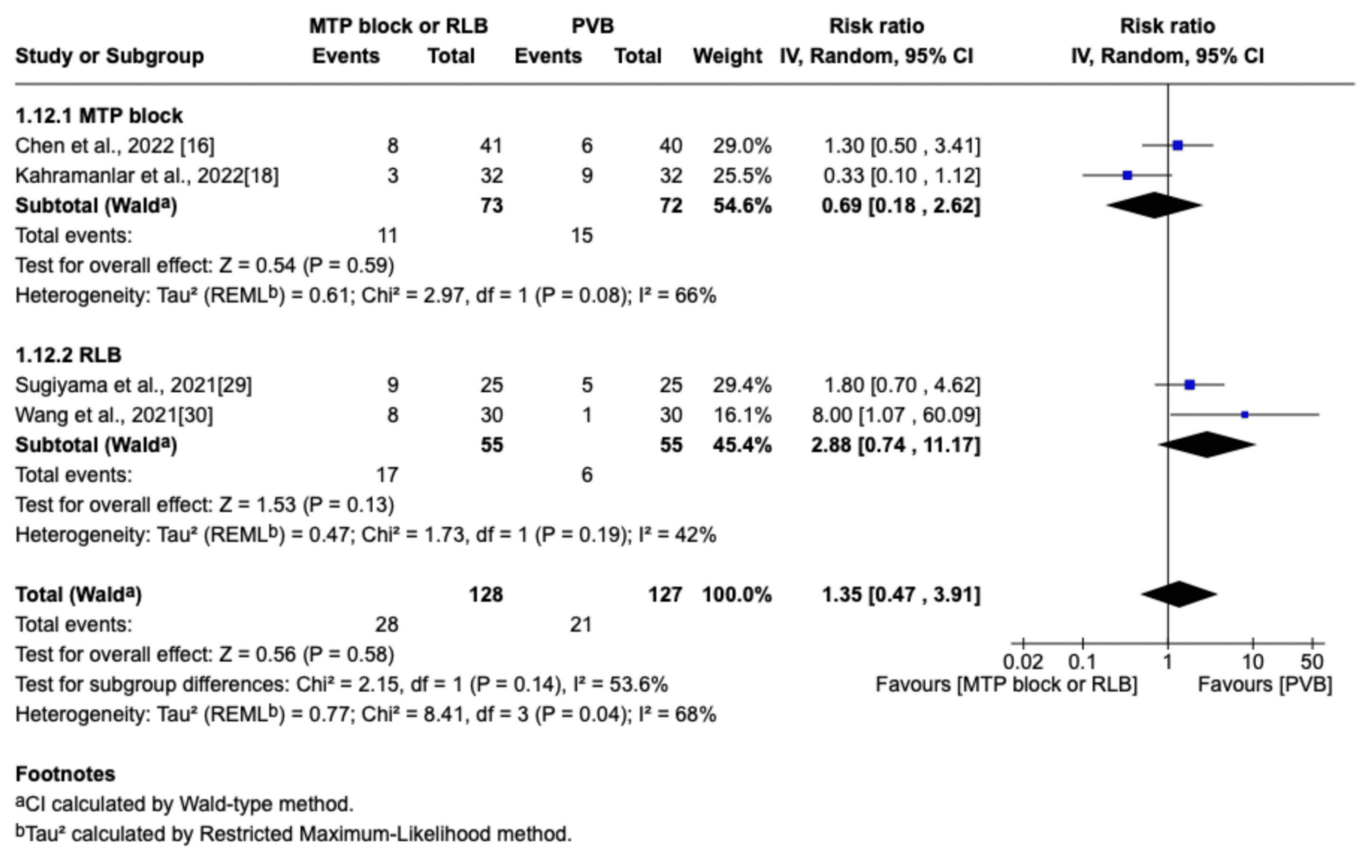
Forest plot of the risk ratio for postoperative nausea and vomiting MTP: midpoint transverse process to pleura; RLB: retrolaminar block; PVB: paravertebral block; MD: mean difference; SD: standard deviation

PONV was reported in four studies [16,18,29,30]. Both groups showed no difference in the incidence of PONV (RR=1.35; 95% CI=0.47 to 3.91; p=0.58; I^2^=68%; Figure 14). The sensitivity analysis indicated that the Kahramanlar et al. (favoring RLB/MTP block) and Wang et al. (favoring PVB) studies were particularly influential on the pooled results [18,30].

Intraoperative Hypotension

**Figure 15:**
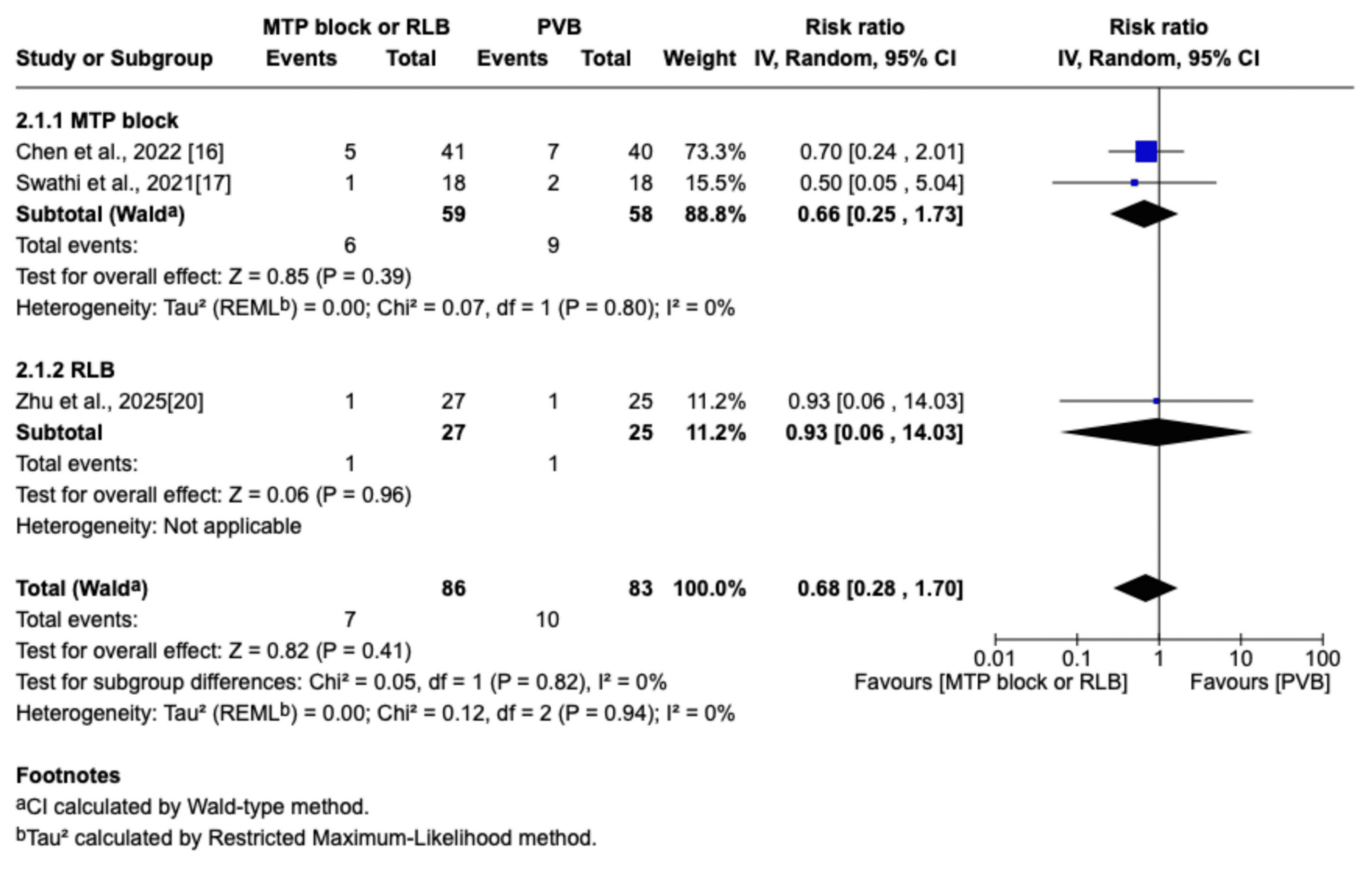
Forest plot of the risk ratio for intraoperative hypotension MTP: midpoint transverse process to pleura; RLB: retrolaminar block; PVB: paravertebral block; MD: mean difference; SD: standard deviation

The definition of intraoperative hypotension varied between studies, including criteria such as a mean arterial pressure (MAP) of ≤65 mmHg or a >20% decrease from baseline. Intraoperative hypotension was directly reported in two studies [17,20] and indirectly reported in one study [16]. Chen et al. described the need for vasopressors during surgery, and we considered that vasopressor administration reflected the occurrence of hypotension [16]. No significant difference was found between the PVB group and the MTP block/RLB group regarding the incidence of intraoperative hypotension (MD=0.68; 95% CI=0.28 to 1.70; p=0.41; I^2^=0%; Figure 15). Intraoperative hypotension is a well-recognized potential complication following PVB. This phenomenon is primarily driven by the block of the thoracic sympathetic chain, which can lead to vasodilation and subsequent hypotension [32]. The lack of a significant difference between the MTP block/RLB and PVB groups in our analysis suggests that the spread of local anesthetic to the sympathetic chain might be comparable, thereby inducing similar hemodynamic effects.

Block-Related Complications

**Figure 16:**
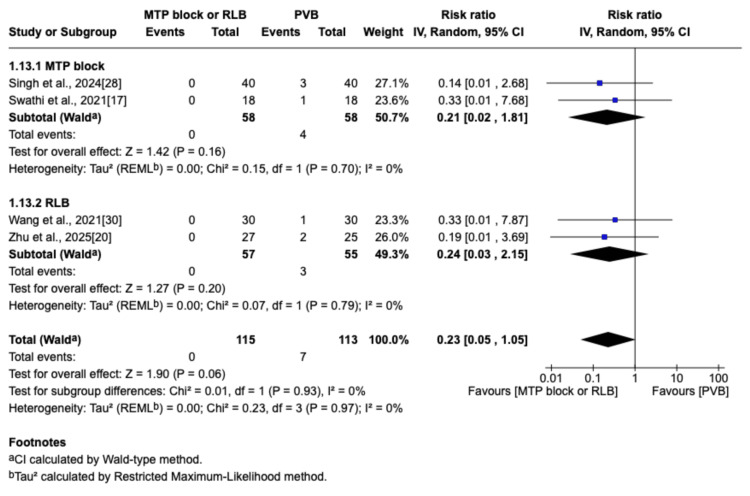
Forest plot of the risk ratio for pneumothorax or pleural puncture MTP: midpoint transverse process to pleura; RLB: retrolaminar block; PVB: paravertebral block; MD: mean difference; SD: standard deviation

Block-related complications were defined and identified based on the criteria used in the included studies. Pleural puncture was defined as needle penetration of the pleura, while pneumothorax and local anesthetic injection into the chest cavity were confirmed under direct thoracoscopic vision. Bleeding complications, including vascular puncture and hematoma, were identified either by the aspiration of blood during the block procedure or by the direct observation of a hematoma via thoracoscopy. Pneumothorax and pleural puncture were actively related by four studies [17,20,28,30], with all seven cases (three pleural puncture reports and four pneumothorax reports) occurring in the PVB group (RR=0.23; 95% CI=0.05 to 1.05; p=0.06; I^2^=0%; Figure 16), resulting in an overall incidence of 2.9% (7/240 patients) in this group. Hematoma was described by Chen et al. as a complication in only one patient in the PVB group [16].

GRADE Assessment

The quality of evidence, based on GRADE for postoperative pain scores at rest and movement at one, six, 12, and 24 hours, was rated from very low to moderate. 

Sensitivity Analysis

We performed a leave-one-out sensitivity analysis for outcomes with heterogeneity >50% to explore the sources of heterogeneity. Sensitivity analysis was performed for postoperative pain scores at rest and during movement (at one, six, and 12 hours) and for postoperative pain, nausea, and vomiting. The statistical heterogeneity (I² statistic) for postoperative pain scores at one hour rest and movement was significantly affected by omitting the study by Kahramanlar et al. [18]. After removing this study, there was no remaining heterogeneity (I²=0). For postoperative pain scores at rest and movement at six hours, the results were significantly affected by omitting Wang et al. [30]. The study by Kahramanlar et al. [18] was particularly influential in postoperative pain scores at rest and movement at 12 hours. 

Subgroup Analysis

Subgroup analysis for MTP block and RLB was performed using a test for subgroup differences in postoperative pain scores at rest and movement at one, six, 12, and 24 hours, cumulative opioid consumption at 24 hours after surgery, time to first postoperative analgesic rescue, PONV, and intraoperative hypotension. There was no significant difference between the subgroups, which allowed us to assume that both blocks could be analyzed together in these outcomes.

Discussion

Summary of Main Findings

This systematic review and meta-analysis of eight RCTs was conducted to determine whether MTP block and RLB are similar to PVB in acute pain management and present reduced block-related complications after thoracic and breast surgeries. We found that PVB was associated with a significant reduction in 24-hour postoperative opioid consumption and superior pain control during movement at 24 hours post-surgery. However, it is important to note that while the difference in pain scores on movement at 24 hours was statistically significant (MD=0.20), the magnitude of this effect is well below the established threshold of 1-2 points required to be considered a minimal clinically important difference for patients [33]. There was no statistically significant difference between the two groups in postoperative pain scores at rest and movement at one, six, 12, and 24 hours. Regarding secondary outcomes, there were no significant differences in the incidence of PONV, time to first postoperative analgesic rescue, time requested to perform block, and intraoperative hypotension. From a safety perspective, all reported cases of pneumothorax and pleural puncture occurred within the PVB group, suggesting an improved safety profile for the MTP block and RLB.

Comparison With Other Reviews

Several meta-analyses and systematic reviews have compared the PVB with other regional analgesia techniques for various surgical procedures, primarily thoracic and breast surgeries [34-37]. Sandeep et al. conducted a network meta-analysis (NMA) that included 38 RCTs involving 2224 patients undergoing VATS to compare different analgesic techniques, including a comparison between the RLB and PVB [38]. In contrast to our study, which found similar postoperative pain scores at 12 and 24 hours (rest and movement), their results indicated that PVB provided lower pain scores than RLB at 24 and 48 hours (rest and coughing) after surgery. In addition, our meta-analysis, which included a larger population for MTP block and RLB, found that there was no statistically significant difference between the RLB and PVB groups for 24-hour postoperative opioid consumption.

In our meta-analysis, we conducted a subgroup analysis to individually compare MTP block or RLB to PVB after thoracic and breast surgeries. The results of our subgroup analysis showed no statistically significant differences between the subgroups. A careful analysis of the four studies contributing to the RLB subgroup revealed significant methodological heterogeneity, which likely explains the conflicting findings and statistical heterogeneity observed in this meta-analysis [19,20,29,30]. While studies by Wang et al. and Sugiyama et al. concluded that PVB provided superior analgesia compared to RLB [29,30], the studies by Zhu et al. and Gao et al. found RLB to be non-inferior or comparable to PVB [19,20]. Wang et al. performed a unique double-level injection for both groups, and Sugiyama et al. potentially introduced a bias by using a less potent anesthetic concentration for RLB (0.25%) compared to PVB (0.5%) [29,30]. Studies have shown that multiple injections result in a more reliable spread than a single injection [39] and that lower concentrations of local anesthetic can achieve similar efficacy in postoperative pain control [40]. In contrast, the more recent studies by Zhu et al. and Gao et al. adopted a single-injection technique and standardized protocols with equal anesthetic concentrations and volumes for both the RLB and PVB groups [19,20]. All four studies were conducted in the setting of thoracic surgery, which makes the comparison more homogeneous but at the same time makes it difficult to extrapolate the results for breast surgery [19,20,29,30].

A further source of methodological heterogeneity among the included trials is the use of local anesthetic adjuvants. In our review, only the study by Singh et al. utilized dexmedetomidine in the study group [28]. It is important to note that this trial did not provide data suitable for pooling for our primary outcome of postoperative pain scores; therefore, its inclusion did not affect those specific results. However, the effect of dexmedetomidine as an adjuvant to PVB is well-documented. A recent meta-analysis by Tang et al. confirmed that adding dexmedetomidine to the local anesthetic for PVB significantly prolongs the duration of analgesia, reduces postoperative pain scores, and decreases 24-hour morphine consumption [41].

A narrative review on RLB corroborated its use in breast surgery and concluded that the published literature suggests that RLB is not inferior to PVB [42]. This review cites a study on patients undergoing mastectomy that found that continuous RLB was not inferior to PVB except for the first 24 hours and was satisfactory [43]. The analysis of the subgroup of the four trials on MTP block included in the present study revealed that the MTP block offers comparable postoperative analgesic efficacy to the conventional PVB [16-18,28]. Although Chen et al. and Singh et al. reported that the MTP block was significantly quicker to perform, this difference was not statistically significant in our meta-analysis [16,28]. Swathi et al. and Kahramanlar et al. both indicated no significant difference in the time to perform the block, and Swathi et al. noted that threading the catheter in the MTP block group was comparatively more difficult [17,18]. Across the four studies reporting pleural puncture or pneumothorax, all seven procedural complications occurred in the thoracic paravertebral block (TPVB) group, resulting in an overall incidence of 2.9% (7/240 patients), while the MTP block and RLB groups had a 0% event rate. This finding, while not statistically significant (p=0.06), contrasts with the 0-0.5% complication rates reported in major retrospective studies on ultrasound-guided PVB by authors such as Niesen et al. and Pace et al. [44,45] and meta-analyses reporting approximately 0.3-0.39% [36,37]. The elevated PVB complication rate in our analysis may be a statistical artifact of smaller sample sizes, where a few events can inflate the incidence rate, or could be related to the learning curve of operators in individual trials. Singh et al. reported a high complication incidence of 7.5% (three cases in 40) in their TPVB group; this single trial accounted for almost half of all adverse events in the meta-analysis, which likely biased the overall pooled incidence [28].

Strengths and Limitations

To our knowledge, this is the first review and meta-analysis directly comparing these "paravertebral by proxy" blocks, ultrasound-guided RLB, and MTP block to classical PVB for thoracic and breast surgery. We exclusively included RCTs to ensure high-quality evidence was obtained. Recognizing significant heterogeneity in study protocols, including variations in injection technique (single vs. multiple), catheter use (vs. single shot), and local anesthetic administration (concentration, volume, adjuvants), we conducted both subgroup (distinguishing between MTP block and RLB techniques) and sensitivity analyses to explore potential sources of this heterogeneity.

However, our analysis has some important limitations. Firstly, only two studies (including one abstract) assessing breast surgery were included, restricting the statistical power and conclusions for this population [18,28]; furthermore, the inherent differences between thoracic and breast surgery mean our pooled analgesic outcomes should be interpreted with caution. Additionally, block performance time data only compared the MTP block to the PVB, preventing definitive conclusions about RLB's performance. Postoperative pain scores were tracked only up to 24 hours, while 48-hour scores and chronic pain outcomes were not assessed. Insufficient standardized data prevented the analysis of the extent of sensory block, and the variability in study protocols in the available literature limited direct comparisons. Only adult patients were included, and no conclusions could be drawn about other specific populations (children, the elderly, and comorbidities). Finally, the inclusion of varied surgical approaches, ranging from different types of VATS (single-port to multiportal VATS) and mini-thoracotomy to mastectomy, introduces considerable clinical heterogeneity and should be considered when interpreting the generalizability of our pooled results.

## Conclusions

The findings of this meta-analysis suggest that both the MTP block and RLB may represent alternatives to the classic PVB for thoracic and breast surgery. While MTP block and RLB provide comparable analgesia during the initial 12 postoperative hours, PVB demonstrates a slight but statistically significant advantage in the later recovery, evidenced by reduced opioid consumption and improved pain management during movement at the 24-hour mark. However, this incremental analgesic benefit is counterbalanced by a significant safety concern, as all reported cases of serious procedural complications, namely, pneumothorax and pleural puncture, occurred in the PVB group. Therefore, the choice of block technique involves a clinical weighting prioritizing maximal 24-hour analgesic efficacy with PVB or the substantially enhanced safety profile and technical simplicity of the MTP block and RLB. Considering the limitations of our findings, including the high degree of clinical heterogeneity and the scarcity of data in the breast surgery population, further studies are required to confirm these benefits, particularly in the breast surgery population, while also standardizing block techniques and assessing long-term pain outcomes.
